# Mitochondrial ATP synthase 8 single-nucleotide polymorphism affects oxidative stress and survival of mice

**DOI:** 10.1007/s00424-025-03123-2

**Published:** 2025-09-20

**Authors:** Gesine Reichart, Johannes Mayer, Tursonjan Tokay, Timo Kirschstein, Falko Lange, Rüdiger Köhling

**Affiliations:** 1 Oscar-Langendorff-Institute of Physiology, Rostock University Medical Center, Rostock, Germany; 2 MSH Medical School Hamburg, Hamburg, Germany; 3 Department of Biology, School of Sciences and Humanities, Nazarbayev University, Astana, Kazakhstan

**Keywords:** Conplastic mouse strains, MtDNA variants, Single-nucleotide polymorphism, MT-ATP8, Oxidative stress, ATP synthase, Ageing, Long-term potentiation, Cognition, Lifespan

## Abstract

Single-nucleotide polymorphisms in mitochondrial DNA (mtDNA) encoded genes of respiratory chain complexes are known to be associated with severe diseases and life-threatening syndromes. In the assembly of the ATP synthase, the enzyme that in the final steps of oxidative phosphorylation generates ATP from ADP and inorganic phosphate, two subunits (ATP6 and ATP8) are mtDNA-encoded. In our study, we investigated the impact of a single-nucleotide polymorphism in *MT-ATP8* with respect to memory function in a preclinical model. Here, we have employed two conplastic mouse strains. The mouse strain C57BL/6 J-mt^AKR/J^ served as a control with wild-type sequence in *MT-ATP8*, while C57BL/6 J-mt^FVB/NJ^ exhibited an m.7778G > T transversion. Using two age groups (3 months and 24 months), levels of reactive oxygen species (ROS), spatial learning in the Morris-Water-Maze, and long-term potentiation were assessed. Immunohistologically, the expressions of NeuN and GFAP were quantified. Additionally, the lifespan of both strains was registered. In comparison to young C57BL/6 J-mt^FVB/NJ^ mice, aged animals had higher ROS levels in the hippocampus. A decreased NeuN/GFAP level was found in C57BL/6 J-mt^FVB/NJ^ mice as well as in old animals of the control strain. Aged animals performed worse in the swimming trials, but no significant differences between both strains were detected. The long-term potentiation recordings revealed reduced synaptic plasticity in young C57BL/6 J-mt^FVB/NJ^ mice. Interestingly, C57BL/6 J-mt^FVB/NJ^ mice presented an extended lifespan compared to animals of the control strain. Together, our data suggest a minor impact of a single-nucleotide polymorphism in *MT-ATP8* on spatial learning and oxidative stress depending on the neuronal tissue. In line with the concept of mitohormesis, our findings may be linked to the longevity of mice harbouring single-nucleotide polymorphisms.

**Supplementary Information:**

The online version contains supplementary material available at 10.1007/s00424-025-03123-2.

## Introduction

The central nervous system (CNS) is one of the most metabolically active organs in the body, with an energy consumption of 20% of the total body’s energy [1]. The main source of energy is ATP, which is produced for the most part in mitochondria by the metabolic pathway of oxidative phosphorylation (OXPHOS). Last in line of the OXPHOS enzyme machinery is the ATP synthase (also referred to as Complex V). Under aerobic conditions, ATP synthase uses the transmembrane electrochemical gradient of protons (proton motive force) across the inner mitochondrial membrane to generate ATP from ADP and inorganic phosphate. Physiologically, the mammalian F_1_F_O_ ATP synthase is composed of 18 different subunits, two of which—ATP6 and ATP8—are encoded by mitochondrial DNA (*MT-ATP6* and *MT-ATP8*, respectively) [2]. Functional ATP synthases are formed by dimerization of those monomers.

In the last three decades, mutations in the mtDNA-encoded subunits ATP6 and ATP8 were identified to be associated with impaired OXPHOS function and mitochondrial diseases [3, 4]. Malfunctions of ATP synthase may lead to oxidative stress, a condition in which reactive oxygen species (ROS) reach cytotoxic levels [5]. Most of the reported mutations with severe effects occurred in *MT-ATP6* or in the mtDNA segment that encodes for both subunits, while *MT-ATP8* variants were sparsely detected. This is not unexpected, since the ATP6 subunit is directly involved in the conduction of protons from the inner mitochondrial membrane to the matrix, whereas the ATP8 subunit links the proton channel and the other subunits of the peripheral stalk [2, 6]. Single-nucleotide polymorphisms of *MT-ATP8* were involved in cardiovascular [7, 8], neurological [9–12], and psychological disorders [13, 14]. *MT-ATP8* and *MT-ATP6* share a sequence overlap of approximately 45 nucleotides. Genetic alterations in this section of the circular DNA affect both subunits of the ATP synthase. Cardiomyopathies were associated with transitions in this overlapping DNA Sect. [6, 15, 16]. In a case report of Imai et al., cardiomyocytes of an infantile patient presented a high level of heteroplasmic mtDNA variant (90% of m8528T > C). In the post-mortem analysis, the protein expression of subunits ATP6 and ATP8 was reduced in the heart and the cells presented an ATP synthase deficiency [15]. Other single-nucleotide polymorphisms affecting the overlap of both genes were accompanied by severe neurological signs [17] or various impairments, including cerebellar ataxia, peripheral neuropathy, and sensorineural hearing impairment [18].

The onset of pathological phenotypes is often linked to the degree of heteroplasmy of the maternally inherited mtDNA variants. So far, no explicit cut-off of the percentage of heteroplasmy has been identified for a decreased OXPHOS function and eventually, for the impairment in physiological functions of the investigated organs. In mice, Hirose et al. reported an impact of mtDNA variants with a degree of heteroplasmy larger than 20% [19]. In humans, a heteroplasmy ranging from 60 to 90% was assumed to be associated with mitochondrial diseases [20].

In our study, we took advantage of two conplastic mouse strains that share the same nuclear background of C57BL/6 J but differ in a single-nucleotide polymorphism (SNP) in a mitochondrial-encoded gene of F_O_ subunit ATP8 (*MT-ATP8*). While the C57BL/6 J-mt^AKR/J^ strain harbours the wild-type sequence (nt7778G), C57BL/6 J-mt^FVB/NJ^ carries a natural SNP (m.7778G > T) that leads to an exchange of aspartic acid to tyrosine [21]. In an acute pancreatitis model, mice harbouring this G-to-T transversion did not suffer from more severe conditions [22], and in a second study, no effects were found on haematopoiesis [23]. However, the animals presented a lower glucose tolerance and impaired beta-cell functions under a high-fat diet in comparison to animals with wild-type ATP8 [24]. So far, the impact of the mtDNA variant on cognition and animal survival has not been investigated. Therefore, aiming to elucidate the impact of this SNP in *MT-ATP8* on the biological phenotype, we exhibited effects on oxidative stress in the brain and cognitive functions. We further investigated whether the genetic alteration may affect the lifespan of the animals, as the concept of mitohormesis [25, 26] suggests that ROS in a narrow physiological range may act as a stimulus to activate the transcription of genes involved in longevity.

## Material and methods

### Conplastic mouse strains and lifespan analysis

All procedures including animal studies were conducted according to national and international guidelines on the ethical use of animals (European Council Directive 86/609/EEC, approval of local authority LALLF in Rostock, Germany (M-V/TSD/7221.3‐1.1‐059/12) and the Animal Care and Use Committee Lübeck (V242‐7224. 122‐5, Kiel, Germany)). All efforts were made to minimise animal suffering and to reduce the number of animals used. Mice were housed in groups of up to four animals per cage under open barrier terms. They were kept under the following stable conditions: room temperature 23 ± 2 °C, relative humidity 40% ± 5%, day‐night rhythm with illumination from 6 AM to 6 PM. Cages were equipped with nesting material (ABEDD, Latvia) and red polycarbonate houses (i.e. environmentally enriched conditions). Water and food (10 mm pellets for murinae (Ratte/Maus-Haltung, ssniff Spezialdiäten GmbH, Germany)) were available ad libitum. For experiments, animals were taken from the housing unit in a randomised fashion to reduce systematic bias. Conplastic mouse strains were generated as previously described in detail [21, 27]. The C57BL/6 J-mt^FVB/NJ^ (referred to as mtFVB; RRID: MGI:4,357,652) strain carries a genetic variant (nt7778 polymorphism) in the *MT-ATP8* gene encoding a subunit of the ATP synthase. The G-to-T transversion at position 7778 results in an aspartic acid-tyrosine exchange. The C57BL/6 J-mt^AKR/J^ (referred to as mtAKR) strain serves as a control. To confirm the mtFVB polymorphism, the mitochondrial genomes of breeders and randomly selected sentinel mice were sequenced by our external partner group of Professor Saleh Ibrahim at the Lübeck Institute of Experimental Dermatology, University of Lübeck. In both strains, experiments were performed with two groups of age: young-adult (3 months ± 10%) mice and aged/senile animals (24 months ± 10%).

Fifty control mice (30 male, 20 female) and 42 mtFVB mice (21 male, 21 female) were used for a longitudinal study to evaluate lifespan. Animals were housed as described above and periodically inspected by certified personnel until natural death. A mouse showing more than one of the following clinical signs was determined moribund [28]: inability to eat or drink; severe lethargy (reluctance to move when gently prodded with forceps); severe balance or gait disturbance; rapid weight loss; an ulcerated or bleeding tumour; enlarged abdomen. Moribund mice were killed, and the age was taken as the best available estimate of their natural lifespan. One mtFVB mouse (1 female) and five control mice (3 male, 2 female) died before day 500 and were excluded from statistical analysis of lifespan differences because their cause of death was likely not due to ageing [29]. In the cohorts of animals whose lifespan exceeded 500 days (mtAKR: *n* = 45, mtFVB: *n* = 41, Table 1), the animals were divided into two groups (natural death or reached humane endpoints) and statistically evaluated. Of those animals, 13% of the mtAKR mice and 47% of the mtFVB mice were euthanised due to humane endpoints. All the other animals died of natural causes.

### Morris-water-maze

To investigate cognitive functions of both mouse strains, we tested the spatial learning and memory performance in an open‐field variant of the Morris-Water-Maze task. All Morris-Water-Maze trials were done in a blinded fashion with respect to the genotype to reduce bias. Before testing the spatial learning and memory performance, the mice were carefully examined for their general state of health. Only animals in good general condition (generally healthy and free of open wounds) were included in the trials. As visual ability is essential for spatial learning in the Morris-Water-Maze, mice were additionally checked for sight disorders [30].

Experiments were performed under constant conditions (room temperature 22 ± 1 °C; water temperature 21 ± 1 °C and brightness (~ 110 lx)) in a separate animal behaviour laboratory with minimal noise. Mice were housed for two days in the animal behaviour laboratory for habituation to the room conditions. To perform the swimming tests, the tank (Ø 110 cm) was filled with opaque water. On each wall of the four quadrants, black symbols (50 × 50 cm) served for navigation. The animals were released from eight possible starting locations. The order of starting locations was randomly determined. To escape the situation, mice had to reach a submerged platform (Ø 7.5 cm, hidden approx. 1 cm below the surface) that was placed in one of the four quadrants.

After the first day of habituation, each mouse was tested in six trials per day on seven consecutive days. Each trial had a time limit of 60 s, followed by 30 s remaining on the platform and resting in a neutral cage for 60 s to allow consolidation. In case of failing to reach the target within the time limit, mice were gently guided to the platform by hand. Testing started at 9:00 am each day. Tracks were recorded with a camera for subsequent data analysis with the software Etho Vision 3.1 (Noldus, Wageningen, Netherlands). Latency and distance measures to reach the platform were recorded for each single trial and calculated as cumulative data of one day for each animal. In case animals did not reach the platform, the latency was set to 60 s. After seven days of training with the platform, an additional probe trial was carried out on day eight. For this purpose, the platform was absent from the tank, and exploration during two trials (30 s each) was monitored. The duration in the four different quadrants (target, opposite, left, and right quadrant) and the frequency to access the target quadrant were calculated.

### Brain slice preparation

Mice were deeply anaesthetised by diethyl ether inhalation (Mallinckrodt Baker, Deventer, Netherlands) and decapitated. The brain was quickly removed and transferred into ice-cold and oxygenated (95% O_2_/5% CO_2_) dissection solution (87 mM NaCl, 25 mM NaHCO_3_, 2.5 mM KCl, 1.25 mM NaH_2_PO_4_, 0.5 mM CaCl_2_, 7 mM MgCl_2_, 10 mM D-glucose and 75 mM sucrose; pH 7.4; osmolality 326–328 mosmol/kg). Next, the cerebellum was removed and the rest of the brain was divided into hemispheres. Hemispheres were sectioned in 400 µm transversal slices for hippocampal formation or 500 µm coronal slices for cortical measurements using a vibratome (Integraslice 7550 MM, Campden Instruments Ltd., UK) in chilled and oxygenated artificial cerebrospinal fluid (aCSF). aCSF was comprised of (in mmol/l) 124 NaCl, 26 NaHCO_3_, 3 KCl, 1.25 NaH_2_PO_4_, 2.5 CaCl_2_, 1.5 MgCl_2_, and 10 D-glucose adjusted to pH 7.4 with an osmolality of 304–312 mosmol/kg. After preparation, slices were transferred into a submerged-type storage chamber for maintenance with oxygenated aCSF and kept for at least 1 h of equilibrium, before starting electrophysiological recordings.

### Long-term potentiation recordings

To assess synaptic transmission and plasticity, field excitatory postsynaptic potentials (fEPSPs) from the Cornu Ammonis area 1 (CA1) region of the hippocampus were recorded. For this purpose, slices were transferred into an interface chamber (BSC-HT, Harvard Apparatus, Holliston, US) maintained at 32 ± 1 °C (TC-10, npi electronic GmbH, Tamm, Germany) and superfused with oxygenated (95% O_2_/5%CO_2_) aCSF (perfusion rate of 2–3 ml/min). Borosilicate glass pipettes (GB150-8P Science Products GmbH, Germany) with a tip resistance of 2–3 MΩ (pulled with PIP5 puller from HEKA Elektronik, Lambrecht, Germany) and filled with aCSF containing an Ag/AgCl wire were used for recordings. Schaffer collaterals were stimulated with a bipolar platinum electrode composed of twisted insulated platinum wire (PT-2 T, Science Products GmbH, Hofheim am Taunus, Germany). Stimulation was controlled by a Master-8 pulse stimulator (A.M.P.I., Jerusalem, Israel) connected to a stimulus isolator (A365, WPI Inc., Sarasota, FL, USA), applying a paired-pulse protocol with a 40 ms inter-pulse interval (IPI) and an inter-stimulus interval (ISI) of 30 s (0.033 Hz). Input–output curves until saturation of the amplitude of fEPSPs were recorded to determine baseline stimulation strength. For further stimulation protocols, the intensity was reduced to half-maximum amplitude. Following 10 min of stable baseline recording, theta-burst stimulation (TBS) was used to evoke long-term potentiation (LTP) of EPSPs. The TBS protocol comprised 3 trains, 20 s apart. Each train consisted of 10 epochs at 5 Hz, containing 5 pulses each (duration 150 µs at 100 Hz). Evoked fEPSPs were amplified and filtered at 1 kHz (EXT-10-2F, npi electronic GmbH, Germany). Recordings were digitised (Micro 1401mkII, CED Ltd., Cambridge, UK) and analysed using Signal 2.16 (CED Ltd.; RRID: SCR_017081). The LTP was calculated 55–60 min after theta-burst stimulation. Slopes of fEPSPs were measured and normalised to the mean of baseline response.

### Mitochondrial superoxide quantification in the hippocampus and neocortex of murine brain slices

To determine oxidative stress ex vivo, superoxide levels as a surrogate for ROS were determined. For this purpose, murine brain slices (see Sect. 2.3 for slice preparation) were exposed to aCSF solution containing 1 µmol/l MitoSOX™ Red (Thermo Fisher Scientific, Waltham, MA, USA) for 15 min at room temperature and protected from light during this procedure. Slices were washed in aCSF for 5 min and fixed in 3.7% paraformaldehyde, cryo-protected with 30% sucrose in 1 × phosphate-buffered saline (PBS) overnight and frozen at −80 °C for storage. To quantify MitoSOX™ Red fluorescence, slices were cut into 10-µm-sections, counterstained, and mounted with ProLong Gold Antifade Reagent containing 4’,6-diamidino-2-phenylindole (DAPI, Thermo Fisher Scientific). At 120 × magnification, regions of interest (ROIs) in the hippocampus Cornu Ammonis area 1 (CA1), CA3 and Dentate Gyrus (DG)) or neocortex (cell layers I/II, III/IV and V/VI) were placed and mean fluorescent signal of MitoSOX™ Red and DAPI were estimated employing microscope Fluoview FV10i (Olympus, Hamburg, Germany). For each subsection within the hippocampus or neocortex respectively, two ROIs were selected and the ratio of MitoSOX™ Red signal and DAPI was calculated to estimate the relative levels of oxidative stress. Data are further merged as the mean of at least six ROIs for each brain section (hippocampus or neocortex). For each animal, five slices of the hippocampal formation and five slices of the neocortex were analysed for MitoSOX™ Red staining.

### Immunohistological analysis

To gauge the ratio of astrocytes and neurons in the hippocampus of the conplastic mouse strains, glial fibrillary acidic protein (GFAP) and neuronal nuclear protein (NeuN) expression were quantified. NeuN is a well-established surrogate marker for neurons [31], while GFAP is primarily found in astrocytes in the CNS [32]. Acute hippocampal slices (see Sect. 2.3 for preparation) were fixed in 3.7% paraformaldehyde, cryo-protected with 30% sucrose in 1 × PBS at 4 °C overnight, and frozen at −80 °C for long-term storage. After cutting 20-µm-thick slices, blocking was performed with 10% bovine serum albumin (SERVA Electrophoresis GmbH, Heidelberg, Germany), 0.05% Triton X‐100 (Merck, Darmstadt, Germany) for 1 h at room temperature. Next, tissue sections were incubated with the primary antibody for rabbit polyclonal anti‐NeuN antibody (Abcam, Cambridge, UK; ab104225 (RRID: AB_10711153); diluted 1:400; 1 h at 37 °C). Sections were washed three times in PBS for 15 min and exposed to mouse monoclonal anti‐GFAP antibody (Abcam; ab10062 (RRID: AB_296804); diluted 1:400; 1 h at 37 °C). Washing was followed by application of secondary fluorescent-labelled antibodies Cy3 goat anti‐rabbit IgG and Cy5 goat anti‐mouse IgG (Thermo Fisher Scientific; a10520 (RRID: AB_10563288) and a10524 (RRID: AB_10562712); diluted 1:2000; 1 h at 37 °C). Finally, the slices were counterstained and mounted with ProLong Gold Antifade Reagent containing DAPI (Thermo Fisher Scientific). Fluorescence analysis was performed by using a laser-scanning microscope (Leica DMI 6000, Wetzlar, Germany) and Leica Application Suite (v. 2.0.0.13332) software (RRID: SCR_013673). For each subsection within the hippocampus (CA1, CA3, DG), two ROIs were selected and the relative expression ratio of NeuN/GFAP was calculated. Data on relative expression are further merged as the mean of all six ROIs for each slice. For each animal, two slices were analysed.

### Statistical analysis

Statistical analysis was performed with SigmaPlot 13.0 (RRID: SCR_003210). Experimental results are presented in box plots or as mean ± standard error of the mean (SEM) for the indicated number of experiments. Before statistical comparison, data were tested for normal distribution and then analysed using tests as indicated. Mean group differences were tested for significance using the nonparametric Kruskal–Wallis test before multiple comparison subgroups were tested with post hoc Dunn’s test or with a parametric One Way Analysis of Variance (with Bonferroni-corrected t-test). For analysis of the Morris-Water-Maze acquisition trial and the comparison of superoxide levels in separate brain sections, a two-way repeated measures ANOVA (Bonferroni t-test) was calculated. To compare superoxide levels in separate brain sections, a two-way ANOVA (Bonferroni t-test) was employed. A paired t-test was used for comparison of LTP recordings between baseline fEPSPs and after theta-burst stimulation. To analyse the lifespan of the animals, a log-rank test was used. A significance level of *p* < 0.05 was considered to be statistically significant.

Cohen’s d was calculated as

$$d=\frac{\mid x_1-x_2\mid}{\sqrt{\frac{\left(n_1-1\right)\times{Var}_1+(n_2-1)\times{Var}_2}{(n_1+\;n_2-2)}}}$$

where *x*, *n* and *Var* denote mean value, number and variance of both groups.

## Results

### Mitochondrial oxidative stress in mtAKR and mtFVB conplastic mouse strains

**Fig. 1:**
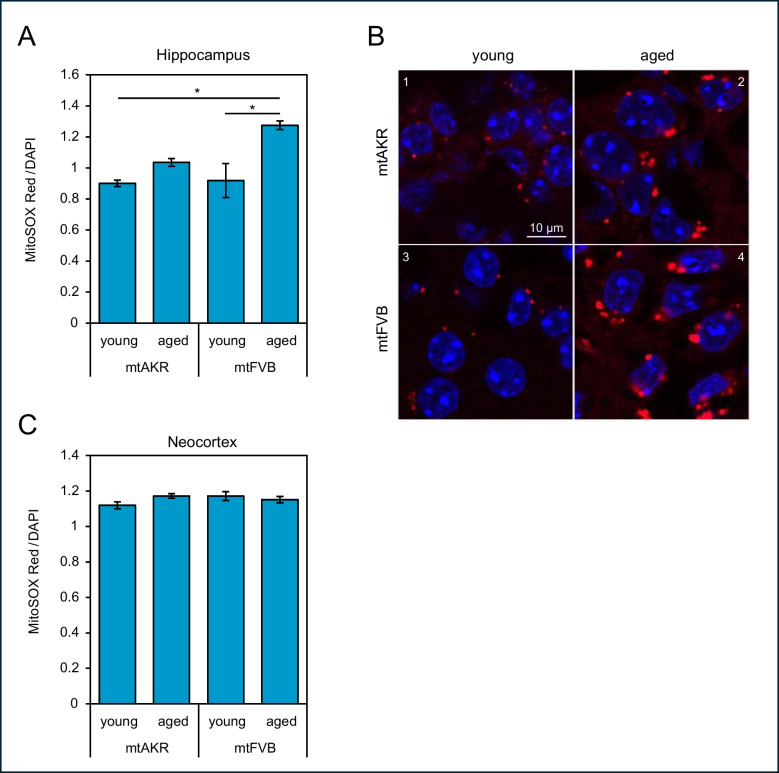
Mitochondrial superoxide levels in brain slices of the hippocampus and neocortex. Superoxide levels in hippocampal and cortical brain slices of mtAKR and mtFVB mice were determined employing MitoSOX Red (shown in red colour). Additionally, nuclei were counterstained with DAPI (blue). **A** The fluorescence level of MitoSOX Red was normalised to the fluorescence of DAPI. Five animals were investigated per group; *p < 0.05 (Kruskal–Wallis test with post hoc Dunn’s test). **B** Representative micrographs were taken from the CA1 region of the hippocampus from young (3 months, micrographs 1 and 3) and aged (24 months, micrographs 2 and 4) mtAKR (1 and 2) and mtFVB (3 and 4) mice at × 120 magnification. Bar represents 10 µm. **C** No significant differences in oxidative stress levels were detected in murine brain slices of the neocortex

This study aimed to explore the impact of a natural SNP in the mitochondrial DNA-localised *MT-ATP8* gene that encodes a subunit of the transmembrane F-type ATP synthase in mice. For this purpose, two conplastic mouse strains, mtAKR (control strain) and mtFVB (mouse strain harbouring the SNP in *MT-ATP8*), were employed. Initially, superoxide levels as surrogate for oxidative stress in young adult animals (3 months ± 10%, referred to as “young”) and aged/senile (24 months ± 10%, referred to as “aged”) were assessed using MitoSOX Red (Fig. 1). In mtFVB (young: 0.92 ± 0.11 vs. aged: 1.28 ± 0.03, 1.39-fold increase, *p* = 0.033) mitochondrial superoxide levels were found to be increased in aged animals in the hippocampus (Fig. 1A; Kruskal–Wallis test with post hoc Dunn’s test). Also, ROS levels were elevated in aged mtFVB mice compared to young animals of the control strain (young mtAKR: 0.90 ± 0.02 vs. aged mtFVB: 1.28 ± 0.03, 1.42-fold increase, *p* = 0.017). Remarkably, in mitochondria of the neocortex, no differences in superoxide levels were detected (Fig. 1C). A two-way ANOVA (factors: strain, mtAKR vs. mtFVB, and brain area, hippocampus vs. neocortex) with Bonferroni post hoc test showed higher ROS levels in the neocortex than in the hippocampal formation (*p* = 0.008). ROS levels did not differ significantly between strains (*p* = 0.105). No significant interaction between mouse strain and brain section was determined.

### Effects of MT-ATP8 single-nucleotide polymorphism on cognitive function

**Fig. 2:**
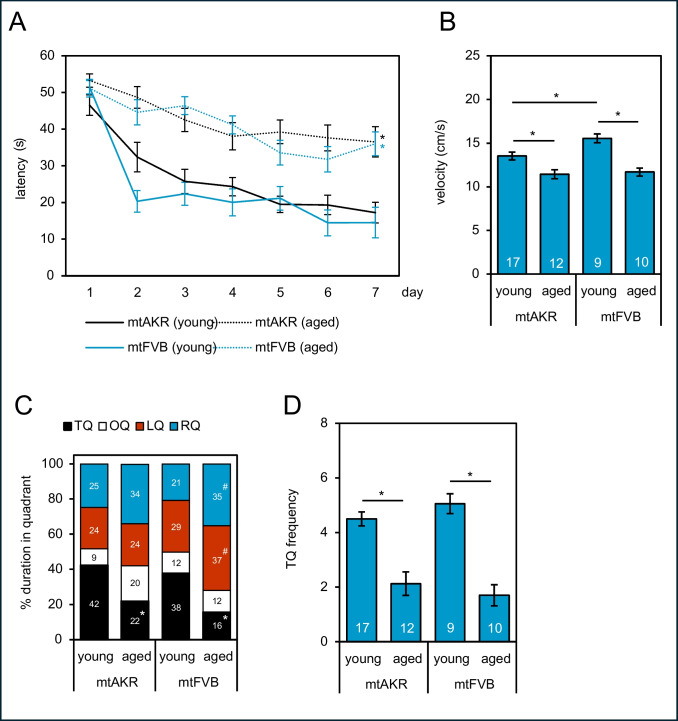
Spatial learning in the Morris-Water-Maze. Mice of the mtAKR and mtFVB strains were challenged in the Morris-Water-Maze. The data represent means ± SEM of **A** the latency of mice to reach the hidden platform (*p < 0.05 (two-way repeated measures ANOVA (Bonferroni *t*-test)) and **B** the mean velocity (*p < 0.05; one-way analysis of variance (with Bonferroni-corrected *t*-test)). The number of mice per group is given in the columns. An additional probe trial was executed on the day after Morris-Water-Maze. **C** In the probe trial, aged mice (24 months) from both strains spent less time in the target quadrant (TQ) than young (3 months) animals (*p < 0.05; two-way ANOVA with Bonferroni *t*-test). The mtFVB cohort exhibited a differential relative swimming duration in left quadrant (LQ) and right quadrant (RQ) in animals of young and old age (^#^*p* < 0.05; two-way ANOVA with Bonferroni *t*-test). No difference in the opposite quadrant (OQ) was detected. **D** Target quadrant (TQ) crossing frequency in the 30-s probe trial; *p < 0.05; one-way analysis of variance (with Bonferroni-corrected *t*-test). The number of mice per group is specified in the columns

Morris-Water-Maze tests were performed in both mouse strains in young and aged animals to assess spatial learning. In both mouse strains, older animals presented a longer latency to reach the hidden platform (Fig. 2A: mtAKR: *p* < 0.001; Cohen’s d = 1.9 and mtFVB: *p* < 0.001; Cohen’s d = 1.8), but no differences between both strains were found (two-way repeated measures ANOVA (Bonferroni t-test)). In both strains, the groups of young animals swam faster than the aged groups (mtAKR: young: 13.5 ± 0.4 cm/s vs. aged: 11.4 ± 0.5 cm/s (*p* = 0.011; Cohen’s d = 3.7) and mtFVB: young: 15.5 ± 0.5 cm/s vs. aged: 11.7 ± 0.5 cm/s (*p* < 0.001; Cohen’s d = 3.8); Fig. 2B). Interestingly, mtFVB mice at 3 months had a small but significantly higher velocity than mtAKR mice at the same age (*p* = 0.034; Cohen’s d = 2.5).

On the day after the Morris-Water-Maze trials, we analysed the duration and frequency of entering the target quadrant in the absence of the hidden platform (Fig. 2C, D). In comparison to aged mice, the cohorts of younger animals spent more time in the target quadrant (mtAKR: 42.5 ± 3.1% vs. 22.0 ± 3.6% (*p* < 0.001; Cohen’s d = 1.3) and mtFVB: 37.9 ± 3.5% vs. 15.8 ± 3.7% (*p* < 0.001; Cohen’s d = 1.7)) and probed the target quadrant more often in the trials (mtAKR: 4.5 ± 0.3 vs. 2.1 ± 0.4 (*p* < 0.001; Cohen’s d = 1.6) and mtFVB: 5.1 ± 0.4 vs. 1.7 ± 0.4 (*p* < 0.001; Cohen’s d = 2.5)). Interestingly, mtFVB mice differed in their relative swimming duration in the left quadrant (3 months: 29.4 ± 3.7% vs. 24 months: 36.9 ± 5.0%, *p* = 0.025; Cohen’s d = 0.6) and in the right quadrant (3 months: 20.7 ± 4.0% vs. 24 months: 35.1 ± 5.2%, *p* = 0.043; Cohen’s d = 1.0). No differences between mtAKR and mtFVB mice within age-matched cohorts were detected.

**Fig. 3:**
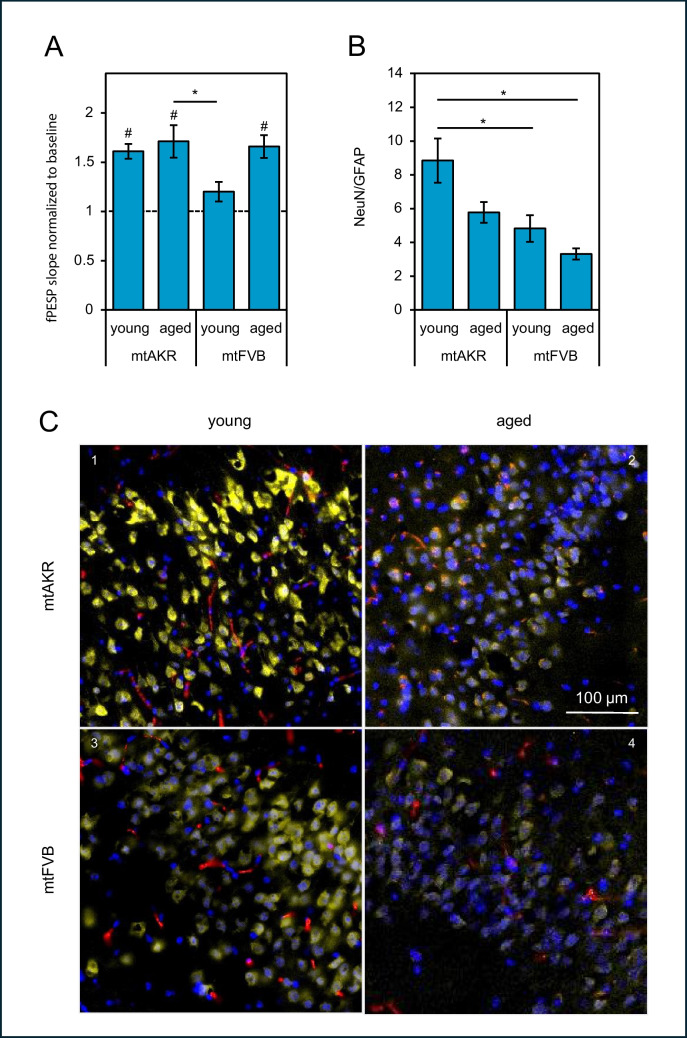
In vitro long-term potentiation (LTP) and neurons/astrocytes distribution of mtAKR and mtFVB mice.** A** In Schaffer collateral-CA1 synapses, field excitatory postsynaptic potentials (fEPSP) of mtAKR and mtFVB mice at young (3 months) and old (24 months) age were evoked (*n* = 5–12 slices based on 4–7 animals per group). Data are shown as means ± SEM of baseline after challenging the slices with a theta-burst stimulation protocol (^#^*p* < 0.05 for fEPSPs of baseline vs. LTP after theta-burst stimulation (Wilcoxon signed-rank test)). Group differences (*p < 0.05) were calculated using Kruskal–Wallis test with post hoc Dunn’s test. **B** The distribution of neurons and astrocytes was assessed by immunological analysis of NeuN and glial fibrillary acidic protein (GFAP) expression in the CA1 region of the hippocampus. The relative fluorescence signals of NeuN and GFAP were determined and the ratio of NeuN/GFAP was calculated. All data are shown as mean ± SEM with *n* = 5–6 animals per group, *p < 0.05 (one-way analysis of variance (with Bonferroni-corrected *t*-test). Young = 3 months old; aged = 24 months old. **C** Representative micrographs were taken from the CA1 region of young (1 and 3) and aged (2 and 4) mtAKR (1 and 2) and mtFVB (3 and 4) mice. NeuN is shown in yellow colour; GFAP in red colour, and nuclei were counterstained with DAPI shown in blue

To obtain more detailed information on hippocampal learning and memory function, LTP recordings as a surrogate for synaptic plasticity in the CA1 region were performed ex vivo in vital brain slices (Fig. 3A). All cohorts except mtFVB at the age of 3 months presented a significant increase of field excitatory postsynaptic potentials after theta-burst stimulation (mtAKR: young (*p* < 0.001), aged (*p* = 0.002) and mtFVB: young (*p* = 0.079), aged (*p* = 0.005), t-test). No differences between young and aged mice in both strains were detected (mtAKR: 1.6 ± 0.1 vs. 1.7 ± 0.2 and mtFVB: young: 1.2 ± 0.1 vs. aged: 1.7 ± 0.1). However, mtFVB mice presented a lower LTP at 3 months in comparison to mtAKR mice at the age of 24 months (mtAKR: 1.7 ± 0.2 vs. mtFVB: 1.2 ± 0.1, *p* = 0.025). In aged mtFVB mice, synaptic plasticity increased to levels comparable to those of the control strain.

Since we found an impairment in the synaptic plasticity in mtFVB mice at the age of 3 months, we asked whether changes in brain tissue composition can be found in this strain. Therefore, the ratio of neurons and astrocytes was estimated by NeuN/GFAP staining (Fig. 3B, C). The mtAKR strain presented a significantly higher NeuN/GFAP ratio than mtFVB at young and old age (young mtAKR: 8.9 ± 1.3 vs. young mtFVB: 4.8 ± 0.8 (*p* = 0.022), young mtAKR: 8.9 ± 1.3 vs. aged mtFVB: 3.3 ± 0.3 (*p* = 0.002), Fig. 3B). However, no significant differences within each strain between young and aged mice were found.

### MT-ATP8 single-nucleotide polymorphism prolongs survival in a conplastic mouse strain

**Fig. 4:**
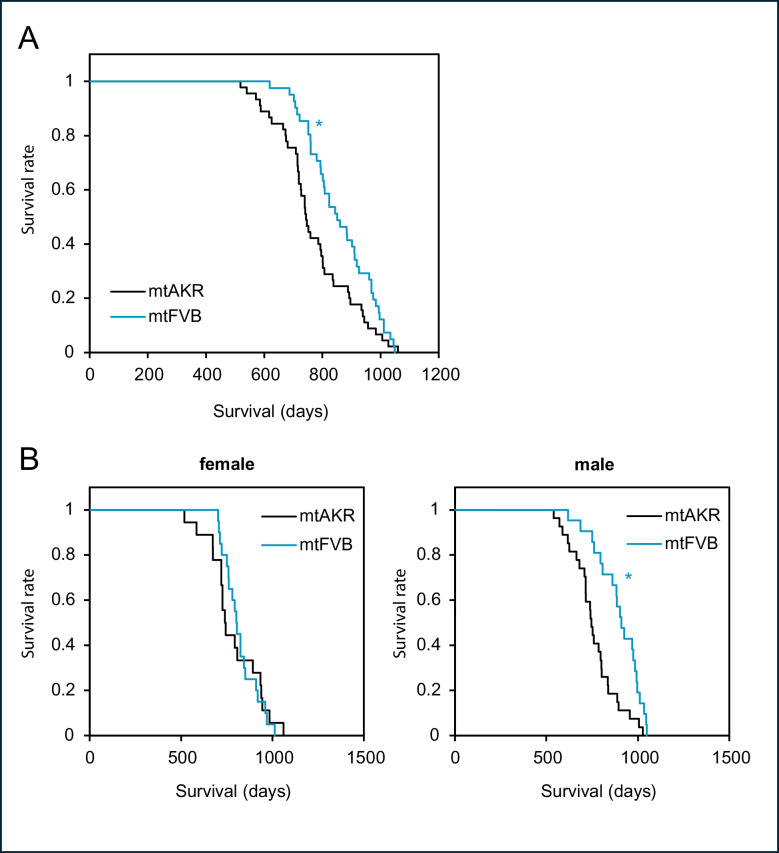
Survival analysis of mtAKR and mtFVB mice.** A** Kaplan–Meier survival curves of mtAKR (*n* = 45) and mtFVB mice (*n* = 41). Mice harbouring mtDNA m.7778G > T polymorphism (mtFVB) demonstrated a longer lifespan than wild-type animals (mtAKR; *p = 0.016; log-rank test). **B** Lifespan analysis of male and female mice of both strains. Intrasexually, the male mtFVB cohort presented a prolonged survival compared to the sex-matched controls (mtAKR (*n* = 27) vs. mtFVB (*n* = 21), *p* < 0.001; log-rank test). No significant difference was found within the female sex (mtAKR (*n* = 18) vs. mtFVB (*n* = 20))

Since mutations in OXPHOS genes of mitochondrial DNA may affect the lifespan [19, 33], we performed a lifespan analysis of wild-type and *MT-ATP8*-mutated mice. A total of 45 mtAKR (27 males and 18 females) and 41 mtFVB (21 males and 20 females) mice were included in a survival analysis. The Kaplan–Meier survival curves are presented in Fig. 4. Control mice of the mtAKR strain had a median survival of 744 days and a significantly shorter lifespan compared to mtFVB mice with a median survival of 851 days (Table 1; log-rank test; *p* = 0.016; Cohen’s d = 0.7). No significant differences between female and male mice of the mtAKR strain were found. In contrast, male mice of the mtFVB strain had a longer lifespan than female animals (log-rank test; *p* = 0.009; Cohen’s d = 0.7). As illustrated in Fig. 4B, an intrasex comparison between both strains revealed a prolonged survival of male mtFVB (*p* < 0.001; log-rank test; Cohen’s d = 0.8), but not of females (*p* = 0.884; log-rank test). As for the lifespan analysis, mice that reached humane endpoints were killed, and the age was taken as the best available estimate of their natural lifespan. We additionally analysed whether there was a difference between mice that were censored versus those that were found dead (survival curves can be found in the supplemental material). While in mtAKR only 13% (6 out of 45) of the animals reached the given endpoints, the number was much larger in mtFVB (46% or 19 out of 41 animals). There was no significant difference in euthanised mice according to sex (mtAKR: 3 male and 3 female; mtFVB: 11 male and 8 female).

**Table 1:** Lifespan analysis of mtAKR and mtFVB mice

Strain	Sex	*n*	Median survival (days)	95% CI (days)	Percentiles		Mean survival ± SEM (days)
					75% (days)	25% (days)	
mtAKR	Male	27	747	725–769	681	835	760 ± 24
	Female	18	739	704–774	719	934	788 ± 34
	Total	45	744	717–771	709	838	771 ± 20
mtFVB	Male	21	911	850–972	808	994	897 ± 27
	Female	20	802	776–828	751	851	820 ± 21
	Total	41	851	773–929	760	969	860 ± 18

## Discussion

In mitochondria, in the cascade of oxidative phosphorylation, not only is ATP generated, but also reactive oxygen species (ROS) as a byproduct. Keeping the balance of ROS production and depletion via non-enzymatic antioxidants and anti-oxidative enzymes like superoxide dismutases, glutathione peroxidases, and catalases is mandatory for the homeostasis and physiological functions of the CNS, including learning and memory function [34, 35]. In our study, we hypothesised that a naturally occurring SNP in a mitochondrial DNA-encoded subunit of the ATP synthase potentially affects the enzyme function and thereby subsequently increases oxidative stress in the CNS. Since an imbalance of ROS is linked with impairment of neuronal functions and neurodegeneration [36, 37], we explored whether spatial learning as a surrogate for cognition was affected by a natural SNP.

In the hippocampus, an age-related increase of ROS in mtFVB mice was found. Interestingly, mitochondrial superoxide levels in the layers of the neocortex of both mouse strains were unaffected by the factors of age or *MT-ATP8* variant. In the conplastic mtFVB model, levels of ROS seem to be highly specific for the investigated tissue or organ. Roolf et al. showed that superoxide levels were reduced in bone marrow cells of 3- and 24-month-old mtFVB mice in comparison to an age-matched control strain [23]. In contrast, in pancreatic beta cells and liver tissue lysates, elevated ROS levels were detected, and at the same time, ATP production was diminished [24, 38]. Higher oxidative stress levels were also found in spleen cells [27] and in an acute liver failure model employing C57BL/6 J-mt^FVB/N^ mice [39]. In accordance with our data on cortical ROS, in isolated pancreatic acini and fibroblasts of the skin, no differences were detected [22, 40]. Furthermore, in mtFVB mice, no differences in the expression and enzyme activity of ATP synthase in liver mitochondria were determined [41].

Since we normalised fluorescence signals of MitoSOX Red to DAPI, we tried to establish a feasible way to compare total superoxide levels between the neocortex and hippocampus. Hippocampal superoxide levels were identified to be slightly lower than those obtained in cortical slices, or in the case of aged mtFVB mice, exhibited the same amount of oxidative stress. Contrary to our observations, a systematic analysis of basal ROS levels in various sections of the CNS of naive 12-week-old Wistar rats detected the lowest amount of radicals in the neocortex, whereas hippocampal ROS production was found to be threefold higher [42]. In comparison to our experiments, Vinokurov et al. oxygenated the ex vivo solution to a much lower amount [42]. Hence, the distinct finding may reflect a differential susceptibility to altered oxygenation of the hippocampus.

Other studies in Wistar rats [43] or C57BL/6 J mice [44] could not demonstrate differences between both areas. One may speculate that a differential expression of anti-oxidative enzymes in the hippocampal formation and cortex of the conplastic strains may contribute to our finding.

We could confirm an age-related decline in the ability of spatial learning in mice [45, 46]. However, increased hippocampal superoxide did not entail a regression of the water maze performances. On the contrary, mice overexpressing catalase showed improved hippocampal learning performance, but no differences in ROS levels were determined [47]. The authors suggested that an altered redox signalling might be responsible for the enhancement in the MWM. So far, the role of ROS in the modulation of synaptic transmission and plasticity is not understood in full detail, and at the current state, the radicals have been identified to contribute in an ambivalent manner to synaptic plasticity [48, 49]. Since we did not check for age-related accumulation of mtDNA mutations, we cannot rule out that aged mice may have accumulated spontaneous mutations [50–52] that affected cognitive performance, and eventually, may have interfered with our analysis on the mtFVB phenotype.

Another finding of our study was that the age-dependent decline in spatial learning was not congruent to lower levels of LTP, a molecular surrogate for hippocampal-induced learning and memory function [53–55]. We examined synaptic plasticity changes related to the mtFVB mitochondrial polymorphism because different groups demonstrated that mitochondrial defects can reduce hippocampal LTP initiation. Here, Weeber et al. [56] and Levy et al. [57] showed that mitochondrial dysfunction reduces hippocampal LTP induction. However, these groups did not use stable SNPs but a knockout, respectively, a chemical-induced mitochondrial failure. In our study, we had expected to see a correlation between spatial learning and synaptic plasticity, perhaps with changes in ROS levels or loss of neurons as a mechanistic cause, but LTP was unaffected in aged mice. Our group already examined other mouse strains with mtDNA polymorphisms in complex I [58] and complex IV [59] of the mitochondrial respiratory chain. The complex I study showed a reduced performance in the Morris-Water-Maze, which goes along with increased production of reactive oxygen species in juvenile mice (3 months) but normal synaptic plasticity. The complex IV study showed an elevated mitochondrial superoxide production and a reduced gene expression regarding anti-oxidative enzymes and mitochondrial fission. These data confirmed a mitochondrial dysfunctional phenotype for aged mutant mice, which on the animal level led to a markedly poor physical constitution when performing the Morris-Water-Maze task. Moreover, the median lifespan of these mice was significantly shorter compared to control animals. But again, LTP was unaffected. Interestingly, a number of studies show that indeed the link between learning behaviour and LTP is far from being a direct one. For example, a review by Lynch [60] lists both studies confirming correlations between in vivo learning and LTP, and others dismissing them. Even inverse relationships have been published; for instance, a study from Huang et al. [61] found reduced learning performance which was associated with increased LTP for a mouse model of ageing. Therefore, our findings regarding in vivo spatial learning and synaptic plasticity measured by LTP are maybe one more part of exceeding evidence for the need to find alternative models for in vitro learning and memory analysis.

In the question of differences in brain cell composition, we analysed the ratio of neurons and astrocytes by NeuN/GFAP staining. Physiologically, we expected to find increasing levels of GFAP signal as astrogliosis is described frequently in the process of ageing [62–64]. Remarkably, no significant differences within each strain between young and aged mice were found. Moreover, the mtFVB strain, which did not show any conspicuous in vivo learning performance, presented a significantly lower NeuN/GFAP ratio, which was apparently attributable to the lower NeuN signal. Therefore, one may speculate that a decreased NeuN signal could not be interpreted as a loss of neurons under all conditions. Although NeuN is still a common marker for post-mitotic neurons, it is now known to be an epitope of the Rbfox3 splicing factor [65]; thus, it may expectably be changed under special conditions in neurons. For instance, Ünal-Cevik et al. described the loss of NeuN immunoreactivity after cerebral ischemia in mouse brains, whereas a surrogate neuronal marker disclosed that these neurons still preserved their integrity [66]. Ogino et al. showed in a mouse model of mild traumatic brain injury a reduction in NeuN-expressing cells, while a counterstaining with NeuroTrace did not demonstrate differences between groups [67]. Both reports indicated a decreasing NeuN immunoreactivity under mild stress conditions in the brain. This may also fit our mtFVB strain with significantly higher superoxide levels in the aged mouse hippocampus, which may reflect a culmination of mildly increased amounts of ROS throughout the lifetime.

Regarding the lifespan of the mtFVB model, we expected a reduction of the average lifetime due to oxidative stress or at least that no impairment occurred [33, 68]. Remarkably, our study revealed an extended lifespan of mtFVB mice. This may even be underestimated in our study, as 46% of the mtFVB mice were censored as moribund and killed during the lifespan analysis, compared to only 13% of the control mice. This might be explained by the concept of mitohormesis [25]. The idea of this concept suggests that exposing mitochondria to challenging (but not overburdening) stressors leads to subsequent protection against higher, usually harmful exposures to similar stressors in the future [26, 69]. This is particularly applicable to the generation of ROS, which results in cellular damage at high levels [70, 71], but at low levels stimulates mitochondrial stress response that can enhance lifespan [72–74]. In our measurements, we found elevated superoxide levels in the hippocampus of aged mtFVB mice, while in the neocortex superoxide levels were slightly (but not significantly) increased in young adult mice. As also other groups described elevated ROS levels for this strain, for example in pancreatic islet cells [24] and in mitochondria isolated from spleen cells [27], mitohormesis may be a plausible explanation for the observed lifetime prolongation.

Both sexes contributed to our lifespan analysis. However, only male mtFVB mice exhibited significantly longer survival than control mice and, in addition, were found to live longer than the females of the same strain. Five decades ago, Kunstyr & Leuenberger reported a longer lifespan for male C57BL/6 mice in a study containing more than 1,000 animals [75]. However, current analyses exhibited mixed results (reviewed in Ref. [76]). As reviewed by Austad and Fischer, there is a great variety among studies reporting males outlived females, females outlived males, and also for virtually absent sex differences [76]. In the end, the authors even summarised that there is no consistent sex difference in mouse longevity. Own investigations based on C57BL/6 mice and mice harbouring a UCP2^−/−^ knockout did not find any differences in the lifespan of males compared to females [28].

To date, no mechanistic approach has been established that provides an explanation for sex-specific lifespan in C57BL/6 mice. The observation of the current study contrasts our analysis of a conplastic mouse model with a natural SNP in cytochrome c oxidase (complex IV). Here, we were able to show that the mtDNA variant led to increased hippocampal superoxide levels in mice of old age, but in contrast to our current investigation, a complex IV variant entails a shortened lifespan [59]. The prolonged lifespan of the mtFVB model is not necessarily due solely to effects on the CNS, since the mtDNA variant is expressed systemically. The leading causes of death in C57BL/6 J mice are neoplasms, particularly lymphomas, sarcomas, and lung cancer [77], located in body regions that were not assessed in our analysis.

In summary, our study contributes to the growing field of analysis on natural mtDNA polymorphisms on organ function and ageing phenotypes. For the first time, a SNP in *MT-ATP8* was explored with respect to ageing and cognition. Our data suggest that the investigated SNP in *MT-ATP8* had only mild effects on oxidative stress and cognition, assessed via long-term potentiation recordings and spatial learning in the MWM. No severe pathological impairments in comparison to age-matched controls were detected. Quite the opposite, due to the slightly elevated oxidative stress in mtFVB mice, one may speculate that the animals may have benefited from longevity based on mitohormetic mechanisms [69, 78].

## Supplementary Information

Below is the link to the electronic supplementary material.

ESM 1 (DOCX 18.8 KB)

## Data Availability

The data presented in this study are available on reasonable request from the corresponding author.
