# Supplementary material for: Mitochondrial ATP synthase 8 single-nucleotide polymorphism affects oxidative stress and survival of mice
Source: Pflugers Arch. 2025 Sep 20;477(11-12):1343–57. doi: 10.1007/s00424-025-03123-2 (PMC12640345; doi:10.1007/s00424-025-03123-2)
Supplement: Supplementary file 1 — (DOCX 18.8 KB) [file 424_2025_3123_MOESM1_ESM.docx]

**Supplementary Material**

**Supplementary Figure 1: Kaplan-Meier survival curves of mice that died by natural cause or were euthanised in moribund conditions.** No significant differences were found within the mtAKR strain (39 natural deaths, 6 euthanised, p = 0.936, log-rank test) or within the mtFVB cohort (22 natural deaths, 19 euthanised, p = 0.738, log-rank test).
